# A facile fluid pressure system reveals differential cellular response to interstitial pressure gradients and flow

**DOI:** 10.1063/5.0165119

**Published:** 2023-09-25

**Authors:** Hao Wang, Jingming Lu, Mitesh Rathod, Wen Yih Aw, Stephanie A. Huang, William J. Polacheck

**Affiliations:** 1 Joint Department of Biomedical Engineering, University of North Carolina at Chapel Hill and North Carolina State University, Chapel Hill, North Carolina 27514, USA; 2 Department of Cell Biology and Physiology, University of North Carolina at Chapel Hill School of Medicine, Chapel Hill, North Carolina 27514, USA; 3 McAllister Heart Institute, University of North Carolina at Chapel Hill, Chapel Hill, North Carolina 27514, USA

## Abstract

Interstitial fluid pressure gradients and interstitial flow have been shown to drive morphogenic processes that shape tissues and influence progression of diseases including cancer. The advent of porous media microfluidic approaches has enabled investigation of the cellular response to interstitial flow, but questions remain as to the critical biophysical and biochemical signals imparted by interstitial fluid pressure gradients and resulting flow on resident cells and extracellular matrix (ECM). Here, we introduce a low-cost method to maintain physiological interstitial fluid pressures that is built from commonly accessible laboratory equipment, including a laser pointer, camera, Arduino board, and a commercially available linear actuator. We demonstrate that when the system is connected to a microfluidic device containing a 3D porous hydrogel, physiologic pressure is maintained with sub-Pascal resolution and when basic feedback control is directed using an Arduino, constant pressure and pressure gradient can be maintained even as cells remodel and degrade the ECM hydrogel over time. Using this model, we characterized breast cancer cell growth and ECM changes to ECM fibril structure and porosity in response to constant interstitial fluid pressure or constant interstitial flow. We observe increased collagen fibril bundling and the formation of porous structures in the vicinity of cancer cells in response to constant interstitial fluid pressure as compared to constant interstitial flow. Collectively, these results further define interstitial fluid pressure as a driver of key pathogenic responses in cells, and the systems and methods developed here will allow for future mechanistic work investigating mechanotransduction of interstitial fluid pressures and flows.

## I. INTRODUCTION

Osmotic and hydrostatic pressure gradients across biphasic tissue drives flow of fluid through the pores in the extracellular matrix (ECM) and across tissue boundaries.^1^ Gradients in interstitial pressure and resulting interstitial flow play a role in a number of developmental and homeostatic processes including lymphangiogenesis^2,3^ and wound healing.^4^ Dysregulation of interstitial pressure and flow contribute to pathophysiological processes,^5^ and elevated interstitial fluid pressure is considered physical hallmarks of cancer.^6^ Yet, how driving pressures and flows at the tissue scale relate to the signals experienced by individual cells remains unclear due, in part, to a lack of accessible experimental systems for recapitulating physiologically relevant, controlled pressures and flows *ex vivo*.

Interstitial flow transports soluble signals via convection while imparting drag forces on embedded cells and the solid phase of the ECM. In seminal work using modified transwell assays and supported by accompanying computational models, Swartz *et al.* demonstrated that convection of chemokines by interstitial flow can direct the migration of tumor cells at intermediate Peclet-numbers due to the establishment autologous chemokine gradients.^7,8^ This transport-mediated signaling has been shown to depend on the kinetics of ECM adsorption of chemokines^7^ and has recently been leveraged to direct angiogenesis and the formation of vascular networks.^9^ The majority of work exploring the role of physical forces arising from fluid pressure and flow within the ECM has been in the context of articular cartilage mechanics, where poroelastic constitutive laws have been found to recapitulate stress–strain responses of cartilage at various length scales.^10,11^ More recently, a number of studies using microfluidic devices that contain poroelastic hydrogels^12^ have suggested that mechanical forces arising from interstitial fluid flow can influence morphogenic processes at the cellular scale, including angiogenesis,^13–16^ lymphangiognesis,^17^ macrophage polarization and migration,^18^ and tumor cell migration.^19–23^ This growing body of work increasingly suggests mechanotransduction of fluid and solid stresses arising from flow through poroelastic media is a driver of myriad cellular processes. However, understanding the nature of forces imparted on cells, how the biophysical properties of cells and ECM modulate these forces, and how these forces compete with soluble signals is challenged by limited control over pressure and flow at the cellular scale.

Broadly, two approaches have been employed to recapitulate interstitial flow within microfluidic devices: (a) the use of a syringe pump to drive a constant volumetric flow rate of fluid into the device and through a hydrogel^24^ and (b) the use of hydrostatic pressure reservoirs to impart an initial fluid pressure gradient across the hydrogel that dissipates with time as fluid flows from the source reservoir to the sink.^25^ Challenging interpretation of results from either approach is the fact that the application of interstitial flow has been shown to upregulate expression and secretion of matrix metalloproteinases that degrade the hydrogel and, thus, alter the resistance to flow over time.^25,26^ Furthermore, there exists an outstanding question as to whether gradients in fluid pressure,^19^ shear stress at the cell surface,^21^ total fluid drag,^22^ or a combination of these signals drives cellular responses. Addressing this question requires systems that can maintain constant fluid pressure gradients even as the resistance to flow changes with time in order to compare the cellular response to those from cells cultured in constant flow systems. Maintaining constant fluid pressure as fluid flows through a porous media with time-varying hydraulic conductivity requires feedback control over applied pressures and, thus, increases complexity of the overall system, possibly at the sacrifice of throughput or yield.

Here, we demonstrate a system for maintaining constant fluid pressure across a hydrogel over multi-day timescales with a pressure resolution of less than 1 Pa. The pressure control system is built from readily available parts, including a laser pointer, camera, commercially available linear actuator, and an Arduino board as a controller. We further implement a microfabrication method that does not require a cleanroom and use commonly available laboratory tools. We then validate fluid flow profiles using a porous media constitutive law in a finite element model of the device. We then test the utility of the constant pressure system by applying a regulated, constant pressure gradient to breast adenocarcinoma cells embedded within a hydrogel and compared the cellular response to cells exposed to a constant volumetric flow rate. The results demonstrate differential ECM remodeling in the two conditions, indicating that the effects of interstitial flow on embedded cells are highly dependent on local pressure and flow profiles.

## II . METHODS

### A. Microfluidic device fabrication

**FIG. 1.**
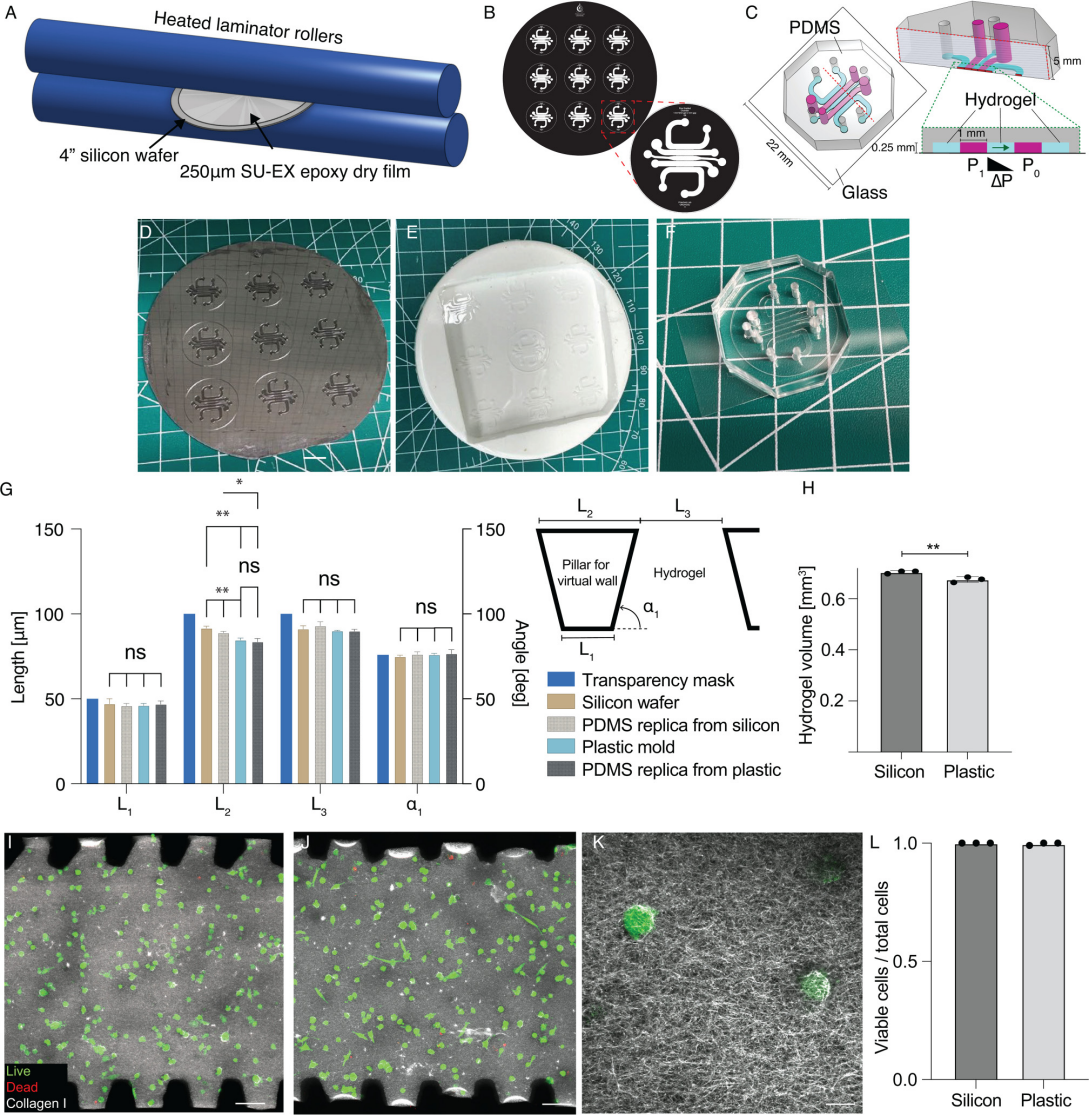
Device fabrication using SU-EX dry films and replica molding. (a) Dry 250 *μ*m SU-8-epoxy thin films (SU-EX) were laminated onto silicon wafers using a commercial laminator with rollers heated to 65 °C and at a speed of 1 ft/min. (b) Film transparency masks for photolithography to pattern (c) five parallel microfluidic channels separated by virtual walls to allow control over interstitial fluid pressure and application of interstitial flow. (d) Resulting silicon master molds were used to fabricate (scale bar 1 cm) (e) plastic master molds for subsequent replica molding of (scale bar 1 cm) (f) PDMS microfluidic devices. (g) Quantification of key geometric properties critical for functioning of the virtual wall. (h) Comparison of total hydrogel volume as measured by confocal microscopy of collagen I hydrogel. Staining of live MDA-MB-231 cells with calcein-AM (green) and dead cells with ethidium homodimer I (red) embedded in collagen I hydrogels (gray) within devices fabricated from (i) silicon master molds and (j) plastic molds (scale bar 100 *μ*m). (k) High magnification imaging demonstrates that live cells are embedded within the collagen hydrogel porous network structure (scale bar 20 *μ*m). (l) Quantification of live cells as a fraction of total cells as determined from confocal micrographs of samples stained with calcein-AM and ethidium homodimer I.

Microfluidic devices containing 3D collagen hydrogels were fabricated from polydimethyl siloxane (PDMS, Sylgard 184, The Dow Chemical Company, Midland, MI, USA), using soft lithography from master molds patterned by photolithography of photoresist-epoxy laminates (Fig. 1). A 40 K dpi (8-*μ*m minimum feature size) film transparency mask [Fig. 1(b), Fineline Imaging, Colorado Springs, CO, US] consisting of five parallel channels separated by trapezoidal posts to form virtual walls^22,27^ was designed to enable three parallel channels filled with collagen hydrogels each flanked with two media channels [Fig. 1(c)]. Silicon wafers (100 mm single-side polish, Test grade, University Wafers, Boston, MA, USA) were cleaned with 70% vol/vol ethanol in water then laminated with 250 *μ*m photoresist-epoxy laminates (SU-EX thick dry films, DJ Microlaminates, Sudbury, MA, USA) using a laminator heated to 65 °C with a rolling speed of 1 ft/min. A post-lamination bake at 70 °C for 15 min then 95 °C for 5 min was used to anneal surface defects and to remove air bubbles. Laminated wafers were next illuminated through the transparency mask with columnated 365 nm light at an energy density of 1000 mJ/cm^2^ for 100 s using a mask aligner (MA6/BA6, Karl Suss, Garching, Germany). A post-exposure bake at 65 °C for 30 min and 95 °C for 2 h was applied prior to development with SU-8 developer (Kayaku Advanced Material, Westborough, MA, USA).

Patterned wafers were treated with air plasma for 2 min (Harrick Plasma, Ithaca, NY, USA) and then passivated by vapor deposition of trichloro-1H-1H-2H-2H-perfluorooctyl silane (Sigma-Aldrich, St. Louis, MO, USA) for 2h. PDMS (Sylgard 184, 1:10, crosslinker: base) was poured onto the mold and cured at 60 °C for 24h. For replica molding, the first casting of PDMS was used as a negative mold for fabricating plastic copies of the silicon wafer. Smooth-cast (Smooth-Cast 310, Smooth-On, Macungie, PA, USA) part A and part B were mixed 1:1 volume ratio and poured onto the PDMS mold. The mixture was vacuumed for 10 min to remove air bubbles prior to curing at room temperature overnight, and the fully cured smooth-cast mold was then used as a master mold for subsequent soft lithography. PDMS was poured onto plastic master molds and cured at 60 °C for 24h. After curing, PDMS was removed from the mold, cut into individual devices, and holes were punched to create gel injection and media ports. PDMS devices and cover glasses (22 × 30 mm^2^, 0.16–0.19 mm thick glass cover slips, Electron Microscopy Sciences, Hatfield, PA, USA) were then treated with air plasma for 30 s prior to bonding and baking at 100 °C for 1h. Next, channels designed to contain collagen hydrogels were treated with 2 mg/ml dopamine hydrochloride (H8502-5G, Sigma-Aldrich, St. Louis, MO, USA) in 10 mm tris-HCl buffer pH 8.5 (bioWorld, Dublin, OH, USA) at room temperature for 2h. The devices were then washed twice with de-ionized (DI) water and baked for 24h at 60 °C. Before each experiment, the devices were sterilized by exposure to UV light for 30 min.

### B. Hydrogel synthesis

Collagen type I hydrogels were synthesized from rat tail collagen as described previously.^28^ Briefly, reconstitution buffer (RB) was made by dissolving NaHCO_3_ and 4-(2-hydroxyethyl)-1-piperazineethanesulfonic acid (HEPES) in DI-H_2_O to a final concentration of 24 mg NaHCO_3_/ml H_2_O and 96 mg HEPES/ml H_2_O and sterile filtered through 0.2 *μ*m syringe filters (MilliporeSigma, Burlington, MA). Dulbecco's Modified Eagle's Medium (10× DMEM, Sigma-Aldrich, St. Louis, MO, USA) was also sterile filtered. To generate sufficient hydrogel volume for five devices, 400 ml 2.5 mg collagen type I/ml total solution was prepared by mixing 22.8 ml of 10× DMEM, 22.8 ml of RB, 7.4 ml of 1 M NaOH, 120.7 ml of PBS, 206 ml of 4.39 mg/ml collagen type I from rat tail (Corning, NY, USA), and 22.8 ml of AlexaFluor 647-conjugated collagen type I (prepared as previously described^29,30^). Collagen pre-polymer slurry was injected into microfluidic devices through gel ports [Fig. 1(c)], and devices were placed in 37 °C incubator for 30 min prior to hydration with PBS or cell culture medium. To limit evaporation, each device was placed in a 60 mm Petri dish (Falcon^™^ Bacteriological, ThermoFisher, Waltham, MA, USA), containing a kimwipe (Kimberly-Clark, Irving, TX) soaked in PBS + 1% (vol/vol) penicillin–streptomycin (ThermoFisher).

### C. Cell maintenance and seeding in microfluidic devices

MDA-MB-231 breast adenocarcinoma cells were acquired through ATCC via the National Cancer Institute Physical Sciences-Oncology Network Bioresource Core Facility and provided as a generous gift by Dr. Imran Rizvi. MDA-MB-231 cells were used from passage 3 to 6. Prior to experiments, the cells were grown in 100 mm Petri dishes in growth media [DMEM low glucose + 10% vol/vol fetal bovine serum (FBS) + penicillin and streptomycin] and passaged at 80% confluency. Cells were lifted with 0.05% trypsin-EDTA, centrifuged at 200× g for 5 min and resuspended to a final concentration of 1 × 10^7^ cells/ml growth medium. For studies in which cells are embedded in collagen hydrogels, PBS was replaced with the appropriate volume of cell suspension to achieve the desired final concentration of cells in total volume of hydrogel slurry. The cell suspension was then carefully mixed with the hydrogel precursor slurry to avoid bubbles, then the mixture was added to the gel channels within the device, and devices were incubated at 37 °C for a total of 30 min. The devices were inverted every 2 min for the first 15 min and every 5 min for another 15 min to ensure a uniform distribution of cells. 50 ml of media was added to the media channels for each device after 30 min, and media was changed daily.

### D. Fluorescence imaging

For fluorescence imaging, cells were fixed using 4% paraformaldehyde (PFA, ThermoFisher, Waltham, MA, USA) at room temperature for 30 min and washed twice with PBS containing calcium and magnesium (PBS++). Cells were permeabilized using 0.3% Triton-X100 diluted in PBS++ for 30 min at room temperature prior to staining with AlexaFluor-488 phalloidin (A12379, ThermoFisher, Waltham, MA, USA) and DAPI (D1206, ThermoFisher, Waltham, MA, USA) diluted in PBS++ (1:200 and 1:1000, respectively). Live vs dead cells were stained prior to fixation with calcein-AM and ethidium homodimer 1 according to kit instructions (Live/DEAD, ThermoFisher). Images were acquired with an Olympus FV3000 laser scanning confocal with 10× U Plan S-Apo, 0.4 NA air objective or 30× U Plan S-Apo, 1.05 NA silicone oil immersion objective. Images were adjusted for brightness and contrast using ImageJ.

To quantify ECM remodeling, AlexaFluor-647 labeled collagen type I was imaged with a 30× U Plan S-Apo, 1.05 NA silicone oil immersion objective on an Olympus FV3000 laser scanning confocal microscope with the same imaging parameters across all conditions. Void fraction and collagen bundling were computed as previously described.^22,31^ Briefly, maximum intensity projections of three slices taken at the medial plane of the collagen hydrogel were generated. The median intensity of each image was computed. Collagen bundling was determined by the number of pixels with intensity values greater than the median value, and each condition was normalized to static control. To determine void fraction, each image was inverted and binarized such that void pixels were assigned a value of 1. Using the same binarization parameters for all conditions, the fraction of positive pixels to total pixels was used to calculate void fraction.

### E. Constant pressure and flow systems

**FIG. 2.**
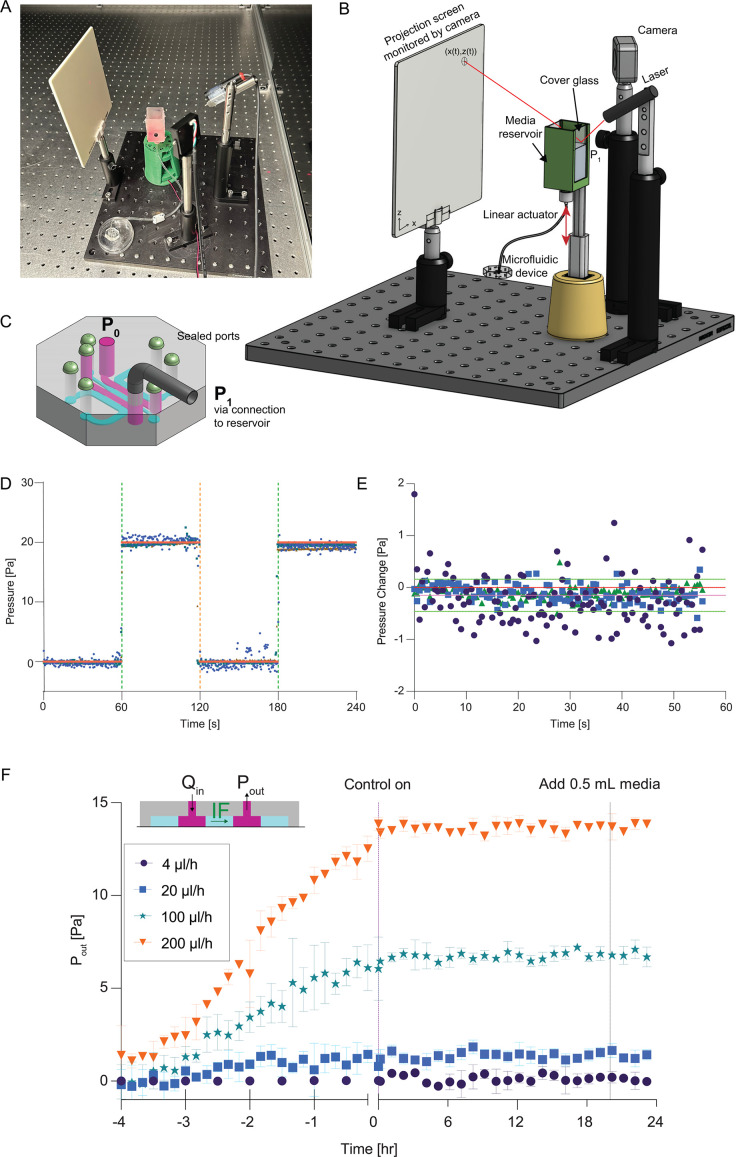
Constant fluid pressure system. (a)—and (b) Image and schematic of key components of the constant fluid pressure system, which adjusts the height of a media reservoir in response to deflections of a laser reflected off the surface of the media reservoir. (c) The constant pressure system is connected to a microfluidic device to establish pressure gradients across hydrogels to recapitulate interstitial pressure and flow. (d) Linear actuator was programmed to move via square wave (±20 Pa with 60 s hold times, red line) and media height was measured (blue markers including three independent experiments) to characterize measurement resolution to physiologically relevant interstitial fluid pressures. (e) Feedback control of linear actuator position was used to maintain constant pressure (different marker shapes indicate independent experiments, red dotted line is the mean position, green dashed line indicates ±1 standard deviation of the mean position, and red dashed line indicated expected position). (f) The constant fluid pressure system was connected to the outlet of the device and used to measure pressure in response to varying inlet volumetric flow rates. With feedback control off (−4 to 0 h), pressure increased linearly with time depending on the flow rate, and with feedback control on (0–24 h) constant pressure was maintained, robust to perturbation including addition of 0.5 ml media directly to the reservoir. Markers and error bars are mean ±standard deviation for ≥3 independent experiments at each flow rate.

To manipulate interstitial pressure and flow, two systems were used. A commercially available syringe pump (Standard Infuse/Withdraw Pump 11 Elite Programmable Syringe Pumps, Harvard Apparatus, Holliston, MA, USA) was used for constant flow rate studies. The constant pressure system was fabricated using a commercially available 3D printer (Form 2, Formlabs, Somerville, MA, USA) and Arduino control board (Arduino Uno Rev3, Arduino, Monza, Italy) to maintain constant fluid meniscus height and hydrostatic pressure in media reservoirs. A compact laser module (PL202, 635 nm, 0.9 mW, THORLABS, Newton, NJ, USA) was used at the light source for measuring the height of the fluid meniscus. The laser was directed toward the surface of a fluid column in a custom fabricated reservoir at an oblique angle. Light reflected by the air–liquid interface was imaged on a white polystyrene viewscreen (EDU-VS1, THORLABS, Newton, NJ, USA) using a camera (Arducam 16 MP, Kowloon, China). The captured image was processed by a custom Python program (supplementary methods) running on a laptop (ThinkPad T14, Lenovo, Hong Kong, China) connected to the camera and the Arduino to determine the center of mass of the laser and to convert this measurement to liquid column height. Based on the height and fluid density, the resulting hydrostatic pressure was calculated. To maintain a constant pressure, the controlling software modulated the center of mass of the laser point within a given range (within ±0.5 pixels, Fig. S1 in the supplementary material) by sending instructions to the linear actuator (Electric Micro Linear Actuator, 12 V, 15 mm/s, USLICCX, China) through an Arduino board to move the reservoir up or down with a step size of 0.1 mm (around 1 Pa) based on the current value. The Arduino board provided a 5 V DC power supply for the linear actuator that leads to a measured movement speed of 3.0 mm/s. The linear actuator was fixed to a 3D-printed base, and the movable end of the actuator was attached to the fluid reservoir via magnets to adjust fluid inlet pressure (Fig. 2). The system was programmed to measure the pressure every 60 s. The time interval was chosen based on the expected flow rate of 20–200 ml/h and the cross-sectional area (576 mm^2^) of the reservoir. The change in pressure is negligible (<1% of total applied pressure of 102 Pa) over a minute (0.0058 Pa to 0.058 Pa).

The custom media reservoir (24 × 24 × 50 mm^3^) was 3D printed (Clear V4, Formlabs, Somerville, MA, USA) using a resin printer (Form 2, Formlabs, Somerville, MA, USA). A threaded luer-lock connector was used to connect the reservoir to standardized tubing and to microfluidic devices. The printed structure was sealed with a cover glass (24 × 50 mm^2^, 0.13–0.17 mm, Cover Glass CS-24X50-100, AmScope, Irvine, CA, USA) and silicone glue (GE Advanced Silicon 2, Rocky Hill, CT) to allow optical access while preventing fluid leakage. Microfluidic devices were connected to the bottom of the reservoir by attaching the tube with connectors to the luer-lock on one end and connecting the other end of the tube to the media port of device using an elbow connector (Fig. 2). The pressure at the inlet of the device is estimated as the hydrostatic pressure calculated using the height difference between the liquid surface in the reservoir and the microfluidic device outlet.

### F. Measurement of hydraulic permeability and interstitial flow

To determine the bulk hydraulic permeability of the collagen hydrogel, the inlet channel to each device was connected to the constant pressure system with feedback turned on to maintain a constant pressure of 400 Pa, and the outlet channel was maintained at 0 Pa. The height of the linear actuator was monitored as a function of time, and the volumetric flow rate was determined to be the rate of change of position of the linear actuator multiplied by the cross-sectional area of the reservoir. Darcy's law was used to determine the hydraulic permeability with the known pressure and flow data, with the viscosity of the medium assumed to be 10^−3^ Pa s.

To measure local fluid velocity vectors in response to an applied pressure, a modified fluorescence recovery after photobleaching (FRAP) method was used.^24^ Fluorescent dextran (70 kDa, FITC-conjugated, ThermoFisher, Waltham, MA, USA) was diluted in PBS to a final concentration of 20 *μ*g/ml, sterile filtered, and added to microfluidic devices containing 2.5 mg/ml collagen type I hydrogel. The inlet channels to the devices were then connected to media reservoirs at fixed heights. Vacuum grease (Z273554, Sigma-Aldrich, St. Louis, MO, USA) was used to seal gel ports, side ports, and tubing connections. Devices were then loaded onto a confocal microscope and the media reservoir was adjusted so that the height differences between the media surface and the device's surface were 3, 4, and 5 cm, respectively, for creating net pressure differences of 300, 400, and 500 Pa across the collagen hydrogel. After establishment of the driving pressure gradient, circular regions were bleached using a 488 nm laser diode to create 124 *μ*m-diameter regions that were imaged every 1 s with a 10× objective (U Plan S-Apo, 0.4 Na, air, Olympus) by confocal microscopy. The velocity magnitude was calculated by the slope of linear fit of the location of the center of mass of the bleached region over time. The location of the center of mass of the spot was tracked through the microscope image using ImageJ. The volumetric flow rate was calculated using the flow velocity at the center of the gel channel multiplied by the cross-sectional area (0.25 × 3 mm^2^). A linear fit of flow rates and the applied pressure was used to calculate the permeability constant of the collagen gel using Darcy's law.

### G. Finite element model

COMSOL Multiphysics (COMSOL, Inc., Burlington, MA, USA) was used to model flow through the collagen hydrogel. The model geometry was imported from the technical drawings of the transparency masks. An extra fine, physics-controlled mesh was used, and results were found to be consistent upon further mesh refinement. The Reynolds number was found to be less than 10^−1^ for all experiments and simulations, and, thus, inertial terms in the Navier–Stokes equation were negligible and Brinkman's equation was used to model flow through porous media. The permeability of the hydrogel was experimentally determined using the location of the linear actuator, and a viscosity of 1.00 × 10^−3^ Pa s was used. All interfaces with PDMS were defined as no-slip boundaries. For pressure-controlled simulations, the fluid pressure at hydrogel–media interfaces in the source channel were set to 294, 392, and 490 Pa, identical to the hydraulic conductivity measuring experiment, while the outlet was held at 0 Pa to match experimental conditions. For flow-controlled simulations, an incoming volumetric flow rate of 20 ml/h was used to match experimental conditions, and the permeability of the hydrogel used in the computation was set to the calculated result from the FRAP assays (3.84 × 10^−14^ m^2^).

## III. RESULTS

### A. Microfluidic device fabrication and replica molding

To fabricate a platform for 3D cell culture under interstitial flow, we laminated 250 *μ*m SU-EX sheets onto silicon wafers [Fig. 1(a)] prior to exposing wafers to UV light through a film transparency mask [Fig. 1(b)] to create a master mold for replica molding individual microfluidic devices with five parallel channels [Fig. 1(c)]. Silicon master molds [Fig. 1(d)] were used as a substrate to fabricate plastic master molds [Fig. 1(e)] for ease of dissemination and archiving of silicon master for preservation. Individual PDMS microfluidic devices were formed using standard replica molding and air plasma-mediated bonding to cover glass [Fig. 1(f)]. The dimensions of the pillars that comprise the virtual wall that separates channels containing hydrogel from those containing media are critical for keeping fluids confined and for the function of the device. We observed a small but significant decrease in the side length of pillars with iterations of molding [Fig. 1(g)], which resulted in a slight but significant decrease in overall hydrogel volume when comparing fabrication directly from the silicon wafer as compared to the plastic mold [Fig. 1(h)]. However, both fabrication methods led to high viability of embedded cells [Figs. 1(i)–1(l)]. Therefore, the plastic molds were used as a substrate for subsequent replica molding.

### B. Constant pressure system

To apply physiologically relevant interstitial flow through the collagen gel confined in the microfluidic device, we developed a system fabricated from off-the-shelf parts incorporating a linear actuator, laser, and camera to provide feedback control for maintaining constant pressure for at least 24h [Fig. 2(a)]. The system was designed to have a small footprint to fit inside standard tissue culture incubators and to be low-cost (total system cost at the time of publication was <$500). The source pressure (P_1_) to the microfluidic device was initially established via hydrostatic pressure in a media reservoir anchored to a linear actuator [Fig. 2(b)]. A laser pointer reflected from the surface of the media to a projection screen was monitored continuously by a camera connected to an Arduino board. The position of the reflected laser was used to adjust the linear actuator position to accommodate pressure head loss via flow through the device (Fig. S1 in the supplementary material). The constant pressure system was then connected to the source media channel within the microfluidic device and the sink channel was either open to the atmosphere or connected to another reservoir depending on the experiment, while all other ports were sealed [Fig. 2(c)].

To demonstrate the function of the constant pressure system, we applied a square wave pressure profile by increasing pressure by 20 Pa, holding for 60 s, then reducing pressure by 20 Pa for 60 s, and repeating. We found that the system maintained pressure in close agreement with the expected profile over multiple independent devices and setups [Fig. 2(d)], with a standard deviation that was <0.5 Pa [Fig. 2(e)]. We then applied a constant volumetric flow rate through a 2.5 mg/ml collagen type I hydrogel within the central channel of the microfluidic device using a syringe pump and measured the pressure at the outlet using the reservoir system developed for maintaining constant pressure. As expected, we found that the outlet pressure increased linearly as a function of time with the slope of the increase determined by the volumetric flow rate [Fig. 2(f)]. With feedback control turned on, the pressure at the outlet remained constant for 24h, robust to perturbations including the direct addition of 0.5 ml (10 Pa) of media to the outlet reservoir [Fig. 2(f)]. Collectively, these results demonstrate that the constant pressure system is capable of maintaining the pressures necessary for sustained, physiologically relevant interstitial flow with velocity magnitudes of 1–>20 *μ*m/s.

### C. Characterization of interstitial flow

**FIG. 3.**
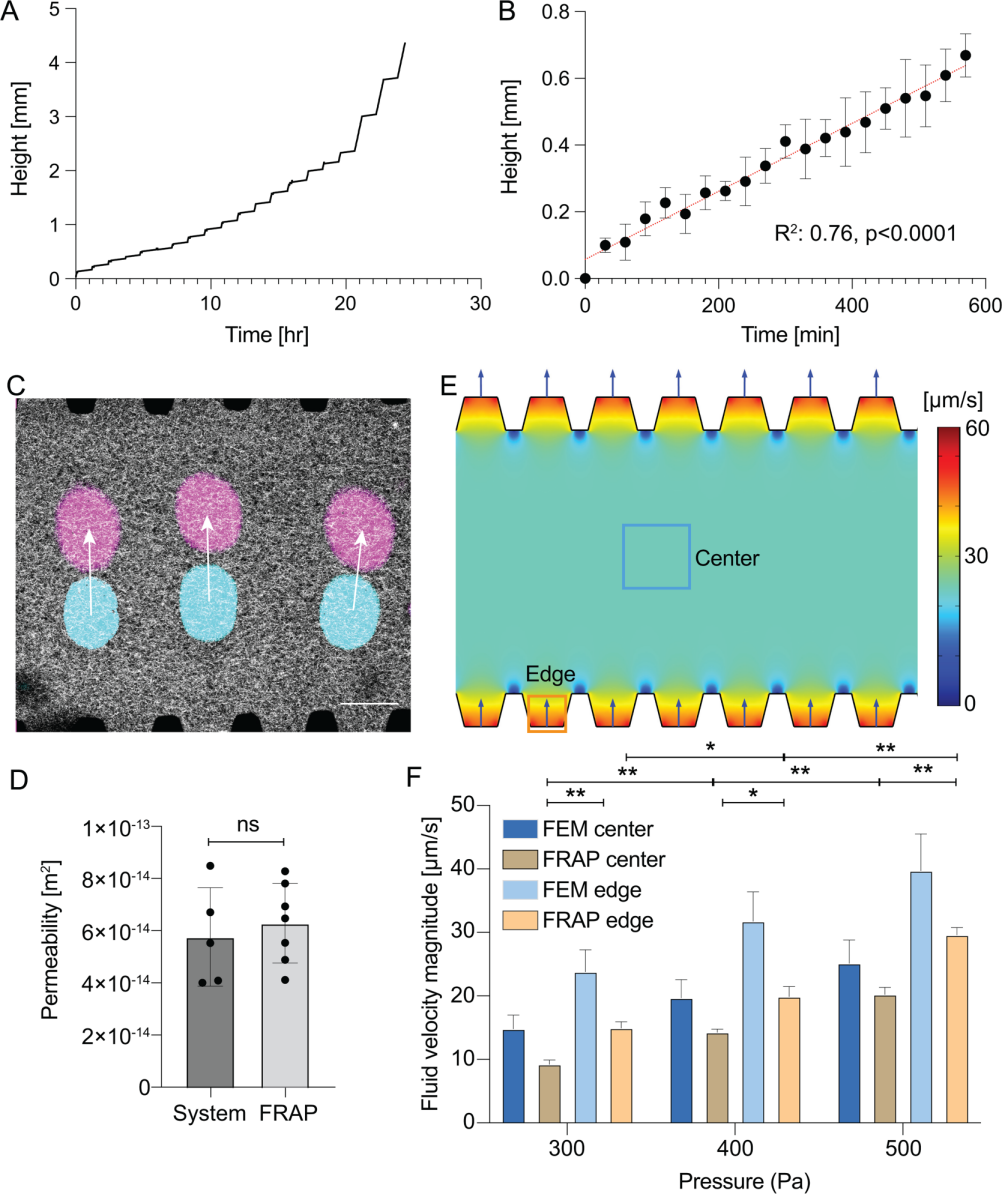
Characterization of interstitial flow. (a) Linear actuator position required to maintain 200 Pa fluid pressure at the inlet as a function of time. (b) Average actuator position for three independent experiments (markers are mean ± standard deviation from n = 3 independent devices, red line indicates linear regression). (c) False colored representative micrographs of 2 MDa fluorescent dextran bleached with 488 nm light at the center of the chamber (teal and magenta spots are inverted bleached regions at t = 0 and t = 11 s, respectively; grayscale is AF647-tagged collagen I) in collagen hydrogel region subject to pressure gradient (350 Pa lower channel and 0 Pa upper channel) to drive interstitial flow. The center of mass of the bleached region was used to compute local flow velocity vectors (white arrow represents velocity vector as measured by tracking center of mass, scale bar 175 *μ*m).(d) Comparison of hydraulic permeability as measured by linear actuator position (system, n = 4) and FRAP methods (n = 7 devices, ns = not significant based on paired, two-tailed t-test). (e) Magnitude of fluid velocity vector field as determined from an FEM model using permeability values from the linear actuator position data. (f) Comparison of experimental and FEM predicted velocity magnitudes measured at the center and edge of the collagen gel region as indicated by labeled regions in (e) (bars are mean ± standard deviation from n = 3 independent devices for FRAP data and n = 5 simulations with each simulation using an independent measurement of hydrogel permeability, statistics compare experimental data with *p < 0.05, **p < 0.01 as determined by two-way ANOVA. No statistical difference was observed between FRAP and simulated velocities for a given location and pressure).

To determine the relationship between fluid pressures applied at the device ports and the interstitial flow velocity field, we first characterized the hydraulic permeability of the hydrogel using the constant pressure system. We connected the inlet of the devices to the constant pressure system with feedback on to maintain 200 Pa applied pressure, and we measured the linear actuator position over time to determine the hydraulic permeability using Darcy's law [Figs. 3(a) and 3(b)]. Using the geometric specifications of the reservoir and collagen hydrogel, we then determined the permeability of the hydrogel to be 5.46 × 10^−14^ m^2^, consistent with previous reports.^24^ To determine local fluid velocities for applied pressures at the device boundaries, we flooded the device with media containing 70 kDa FITC-conjugated dextran and applied 300, 400, and 500 Pa pressure (using hydrostatic pressure as an initial condition with water height difference of 3, 4, and 5 cm) at the inlet and used a modified FRAP technique to measure the interstitial fluid velocity within the hydrogel at the center and edge of the device (Fig. 3). Using fluid velocity magnitude data from seven independent devices and Darcy's law, we found good agreement between the hydraulic permeability measured with FRAP and the bulk measurements from the linear actuator position data [Fig. 3(d)]. Using the hydraulic permeability calculated from the linear actuator data, we then developed a finite element model to determine the fluid velocity field within the hydrogel using Brinkman's equation and found that while the fluid velocity magnitude varies between the posts used to form the virtual wall, the velocity magnitude is uniform in the area of the hydrogel spanning between the posts [Fig. 3(e)]. We then compared the local fluid velocity magnitudes at the center and edges of the device as measured by FRAP to those determined by the FEM using permeability values determined from the pressure feedback system [Fig. 3(f)].

### D. Effects of constant interstitial pressure vs constant interstitial flow on MDA-MB-231 cells

**FIG. 4.**
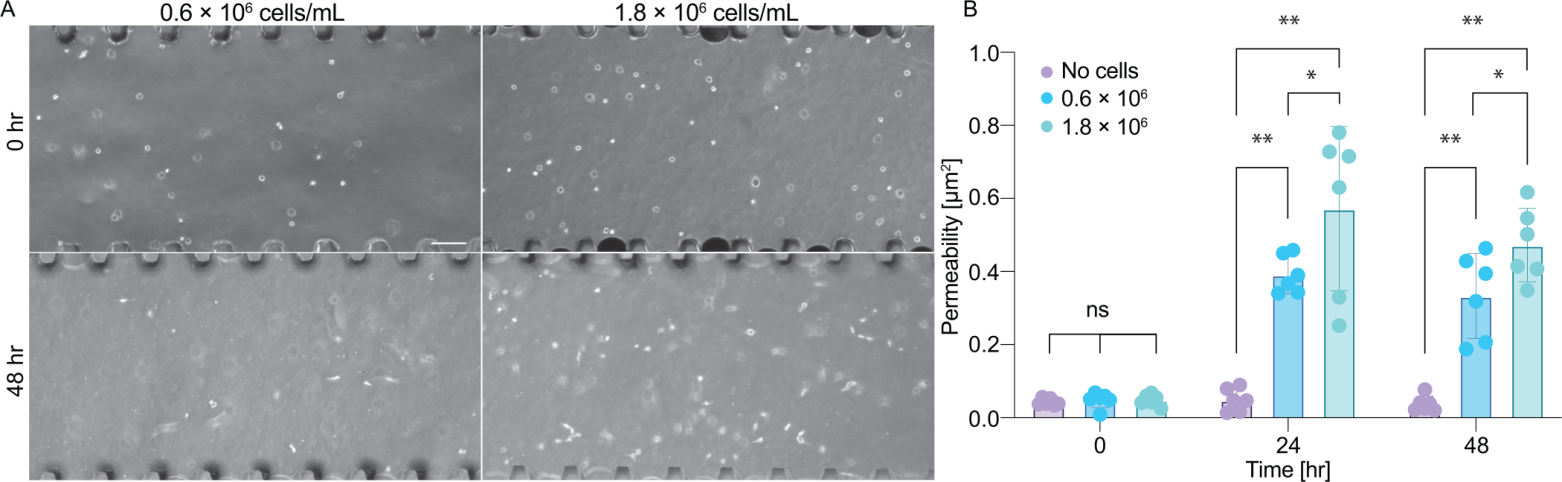
Effects of MDA-MB-231 on collagen hydraulic permeability. (a) Phase contrast images of MDA-MB-231 cells embedded in collagen hydrogels within microfluidic device (scale bar 100 *μ*m). (b) Longitudinal data of hydraulic permeability of collagen as measured by FRAP (n = 6 independent devices for each cell density, error bars represent standard deviation, *p < 0.05 and *p < 0.01 as determined by unpaired two-tailed t-test).

Endothelial and breast cancer cells have been shown to degrade collagen hydrogels in response to interstitial flow.^22,26^ To determine the effects of MDA-MB-231 breast cancer cells on hydrogel permeability, we seeded cells at two different densities (0.6 × 10^6^ and 1.8 × 10^6^ cells/ml) in 2.5 mg/ml collagen I hydrogels [Fig. 4(a)] and measured the permeability of the hydrogel (using FRAP by applying a pressure of at 50 Pa) over time. We observed a significant increase in the permeability within 24h, increasing by 8- and 12-fold, respectively, at the two cell densities [Fig. 4(b)]. These data demonstrate that for an applied constant pressure gradient, the resulting flow rates will increase significantly as the embedded cells remodel and degrade the matrix.

**FIG. 5.**
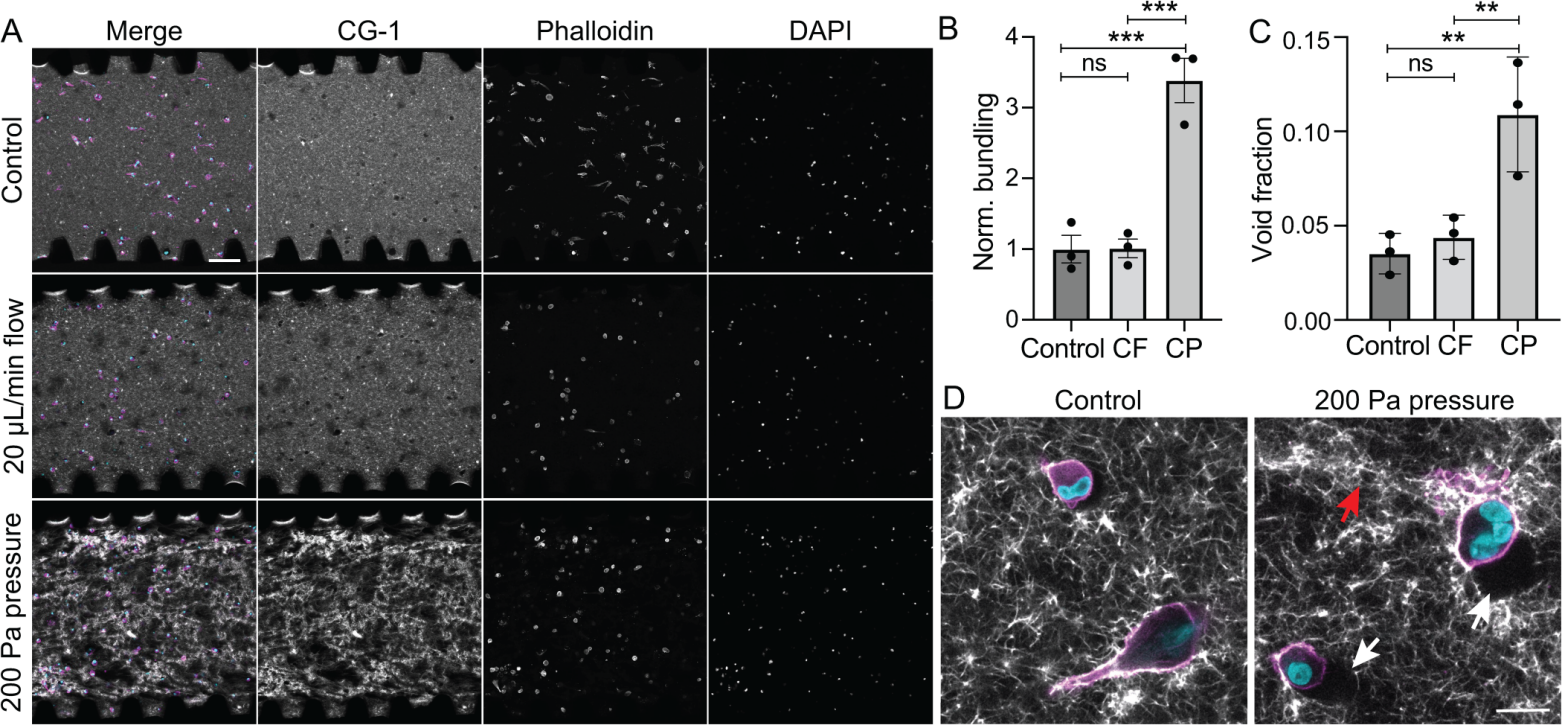
Effects of constant pressure and constant flow on MDA-MB-231 cells. (a) Fluorescent micrographs of cells seeded at 1.8 × 10^6^ cells/ml in fluorescently labeled collagen hydrogels. Images are maximum intensity projections of confocal z-stacks taken after exposure to flow, pressure, or control conditions for 24 h (for merged images, gray = collagen type I, magenta = phalloidin, cyan = DAPI, scale bar is 100 *μ*m). (b) Normalized fibril bundling as measured from fluorescent collagen micrographs. (c) Void fraction as determined from maximum intensity projections of three slices at the midplane of the device (n = 3 independent devices, error bars represent standard error, **p < 0.01, ***p < 0.001 as determined by unpaired, two-tailed t-test). (d) High magnification images demonstrate significant remodeling of the collagen hydrogel under constant pressure conditions (red arrow shows bundling of collagen fibrils and white arrows indicate void areas, scale bar is 20 *μ*m).

To determine whether the time-dependent increase in hydrogel permeability impacts the response to interstitial flow, we applied a constant volumetric flow rate of 20 *μ*l/h to 2.5 mg/ml collagen I hydrogels containing 1.8 × 10^6^ MDA-MB 231 cells/ml by attaching the inlet media channel of the microfluidic device to a syringe pump. In another set of devices, we attached the inlet to the constant pressure system to maintain 200 Pa pressure at the inlet for 24h. These conditions were chosen to induce interstitial flow with a fluid velocity magnitude of 7.4 *μ*m/s at the initiation of the experiment. Interestingly, we observed that while the number of cells remained constant in all conditions [Figs. 5(a) and S3 in the supplementary material], collagen remodeling increased significantly in the constant pressure condition as evidenced by increased fibril bundling [Fig. 5(b)] and in the area void of fluorescently labeled collagen [Fig. 5(c)]. At higher magnification, we observed that the pericellular matrix included regions of increased fibril bundling and void formation in the constant pressure condition [Fig. 5(d)].

## IV. DISCUSSION

Here, using readily available equipment, we introduce a system that is able to measure and maintain fluid pressure with sub-Pascal resolution. We connect the system to a microfluidic platform containing a 3D collagen I hydrogel to recapitulate interstitial fluid pressure gradients and flow. Using an off-the-shelf linear actuator with an Arduino to enable feedback control, we are able to maintain a constant fluid pressure gradient even as highly aggressive MDA-MB-231 cells degrade and remodel the collagen hydrogel and increase the hydraulic permeability. We then implement the constant pressure system to compare the response of MDA-MB-231 cells to a constant fluid pressure gradient with the response to the constant volumetric flow rate.

We found that cancer cells remodel the ECM through degradation and bundling of collagen fibrils. This ECM remodeling changes the hydraulic permeability of the ECM and the resistance to interstitial flow over time, and we found that maintaining a constant interstitial fluid pressure gradient across the hydrogel increased ECM degradation and fibril bundling compared to constant interstitial flow. In this study, collagen type I is used as a model for interstitial matrix, and collagen degradation primarily occurs through expression and activity of matrix metalloproteinases (MMPs).^32^ Interestingly, interstitial flow applied to smooth muscle cells embedded in collagen type I matrices and endothelial cells embedded within fibrin matrices was shown to significantly upregulate expression of MMP-1,^25,33^ which degrades collagen type I,^32^ suggesting that increased ECM degradation via MMPs is a generally conserved mechanism for ECM remodeling in response to interstitial flow. The ECM bundling observed here is consistent with previous experiments with fibroblasts exposed to interstitial flow that demonstrate an initial increase in ECM hydraulic permeability followed by a reduction in permeability that is accompanied by ECM contraction,^34^ and cell-generated traction forces have been shown to drive collagen fibril alignment and bundling in more complex tissues.^35,36^ These results suggest that matrix mechanical properties could play a critical role in mediating ECM remodeling that is crucial to morphogenic events induced by interstitial flow by modulating the resistance to cell-mediated changes in ECM pore size and distribution.^37^ However, future work is necessary to understand how changes in ECM mechanical properties that occur concomitant with changes to interstitial pressure and flow during development and disease pathogenesis govern cell responses.

In solid tumors, increased intratumoral fluid pressure arises from leaky tumor vasculature, a collapse of draining lymphatics, osmotic pressure, and solid stress.^6,38–40^ The relationship between the fluid pressure gradient and resulting interstitial flow is dependent on the hydraulic permeability, which is related in part to the pore size of the solid phase.^41^ Importantly, the total drag imparted on a sphere embedded in a porous medium is the sum of the drag force due to pressure drop across the sphere and the viscous shear stress at the sphere surface.^42^ For porous materials, the force due to shear stress scales inversely with the hydraulic permeability, and the force due to pressure drop scales inversely with the hydraulic permeability squared.^22^ Therefore, the hydraulic conductivity and, thus, the pore size of the ECM modulates not only the total stress imparted on embedded cells but also the relative magnitude of the stress due to pressure drop and stress due to viscous shear. Previously, we showed that interstitial flow can activate integrin signaling in MDA-MB-231 cells and direct cell migration via tension in cell-matrix adhesions,^22,23^ while others have demonstrated a role for the glycocalyx in sensing the shear stress component of interstitial flow.^21^ Together, these studies suggest the ECM pore size and changes to the pore size driven by MMP activity could govern differential signaling responses of embedded cells and underlie the differential response to constant interstitial fluid pressure and constant interstitial flow observed here. Future studies investigating the role of ECM pore size in modulating integrin- and glyocalyx-mediated cell responses are necessary to better understand how ECM biophysical properties modulate effector signaling pathways in the response to interstitial flow.

A growing body of work has begun to explore the use of interstitial fluid pressure gradients and flow in directing morphogenic processes, particularly in the vascular endothelium.^13–16,25,26^ Such an approach is attractive for tissue engineering, as the growth of blood vessels through the establishment of molecular gradients is cumbersome and involves careful balancing of multiple driving soluble cues,^43,44^ the transport of which is not easily controlled in complex tissues.^45^ Thus, low-overhead systems, such as the one described here, for maintaining pressure gradients that can induce angiogenesis and blood vessel remodeling provide an additional tool for future work to grow larger, more complex engineered tissues.

## V. CONCLUSION

In this work, we demonstrate a low-overhead system to maintain constant interstitial fluid pressure with sub-Pascal resolution. We further advance laminate-based fabrication of a 3D microphysiological tumor model that enables microfabrication in standard biology laboratories without the use of complex equipment such as a mask aligner. Using the system, we demonstrate that breast cancer cells rapidly remodel the microenvironment and modulate the resistance to interstitial flow. We show that breast cancer cells respond to a constant pressure stimulus by increasing matrix remodeling as compared to the response to static conditions or constant interstitial flow. Together, these data and the system advanced here will enable future studies to more rigorously investigate how physiological and pathophysiological pressure gradients and flow impact cellular processes including tumor progression and vascular morphogenesis.

## SUPPLEMENTARY MATERIAL

See the supplementary material for details on the controller software, algorithm for feedback control, and data regarding the effects of constant pressure and constant flow on cell count.

## Data Availability

The data that support the findings of this study are available within the article and its supplementary material. Raw images or other raw data are available from the corresponding author upon reasonable request.
